# Hyperhomocysteinemia and hypertension exacerbate cerebrovascular dysfunction and Alzheimer’s Disease pathology in the Tg2576 mouse model of amyloidosis

**DOI:** 10.1002/alz.090484

**Published:** 2025-01-03

**Authors:** Ashley M Carey, Silvia Fossati

**Affiliations:** ^1^ Lewis Katz School of Medicine, Temple University, Philadelphia, PA USA; ^2^ Alzheimer’s Center at Lewis Katz School of Medicine, Temple University, Philadelphia, PA USA

## Abstract

**Background:**

Recent evidence suggests that cerebrovascular dysfunction may precede and contribute to amyloid beta‐(Aβ)‐mediated pathology in Alzheimer’s Disease (AD), particularly promoting endothelial cell damage and stress, causing the cerebral blood flow impairments, cerebral hypoperfusion, and blood brain barrier (BBB) permeability that are pathologically characteristic in AD. Studies have emerged suggesting a link between cardiovascular diseases and AD pathology, showing that cerebrovascular/cardiovascular risk factors (CVRFs), including hyperhomocysteinemia (Hhcy) and hypertension (HTN), and the cerebral consequences of these CVRFs, such as cerebral hypoperfusion, contribute to AD pathology and risk. Despite this, the underlying molecular mechanisms for these associations remain unclear. Previously, our lab has demonstrated that combined exposure of human cerebral microvascular endothelial cells (HCMECs) to Aβ40‐Q22 (vasculotropic Dutch mutant) and homocysteine resulted in potentiated increases in apoptosis and decreases in barrier integrity and angiogenic capabilities. Additionally, we have demonstrated that Aβ in combination with oxygen glucose deprivation exacerbates HCMEC cell death, barrier dysfunction, and angiogenic impairment. We tested the hypothesis that Tg2576 AD mice with hyperhomocysteinemia and/or hypertension will demonstrate exacerbated vascular cell death, BBB dysfunction, angiogenic defects and worsened AD pathology.

**Method:**

WT and Tg2576 AD mice (Swedish APP mutation) were fed with a hyperhomocysteinemia‐inducing diet, HTN‐inducing water (L‐NAME), or both starting at 5 months. Mice were sacrificed at 13‐14 months and tissue was harvested. Apoptotic mediator expression and vascular caspase‐3 activation were measured to assess apoptosis. BBB‐associated tight junction protein expression, GFAP/IBA1 expression and morphology (neuroinflammation), and microhemorrhages were measured to assess BBB integrity. Angiogenic mediator expression and CD31+ microvessel density were utilized to measure cerebral angiogenesis. Soluble and insoluble Aβ42 and Aβ40 were measured to assess AD pathology progression.

**Result:**

Tg2576 mice with CVRFs revealed exacerbated cerebral caspase‐3 activation and apoptosis as well as potentiated BBB and angiogenesis impairment versus WT mice and Tg2576 mice without CVRFs. Additionally, Tg2576 CVRF mice trended towards potentiated increases in cerebral soluble Aβ40 and fibrillar Aβ42.

**Conclusion:**

CVRFs potentiate increases in specific cerebrovascular and angiogenic deficits in Tg2576 mice. This study revealed new molecular mechanisms and druggable targets through which amyloidosis, Hhcy and HTN additively act to produce vascular damage and dysfunction in AD.

